# Evaluating clinical meaningfulness of anti-β-amyloid therapies amidst amyloid-related imaging abnormalities concern in Alzheimer’s disease

**DOI:** 10.1093/braincomms/fcae435

**Published:** 2024-12-03

**Authors:** Manal Aljuhani, Azhaar Ashraf, Paul Edison

**Affiliations:** Radiological Science and Medical Imaging Department, College of Applied Medical Sciences, Prince Sattam Bin Abdulaziz University, Al-Kharj 11942, Saudi Arabia; Division of Neurology, Department of Brain Sciences, Faculty of Medicine, Imperial College London, London W12 0NN, UK; Division of Neurology, Department of Brain Sciences, Faculty of Medicine, Imperial College London, London W12 0NN, UK; Division of Psychological Medicine and Clinical Neurosciences, School of Medicine, Cardiff University, Wales CF24 4HQ, UK

**Keywords:** Alzheimer’s disease, amyloid-related imaging abnormalities, anti-β-amyloid antibodies, clinical meaningfulness

## Abstract

Alzheimer’s disease is the most prevalent form of dementia in the elderly, which is clinically characterized by a gradual and progressive deterioration of cognitive functions. The central and early role of β-amyloid in the pathogenesis of Alzheimer’s disease is supported by a plethora of studies including genetic analyses, biomarker research and genome-wide association studies in both familial (early-onset) and sporadic (late-onset) forms of Alzheimer’s. Monoclonal antibodies directed against β-amyloid demonstrate slowing of the clinical deterioration of patients with early Alzheimer’s disease. Aducanumab, lecanemab and donanemab clinical trials showed slowing of Alzheimer’s disease progression on composite scores by 25–40% based on the measure used. Anti-β-amyloid antibodies can cause side effects of bleeding and swelling in the brain, called amyloid-related imaging abnormalities. Amyloid-related imaging abnormalities typically occur early in treatment and are often asymptomatic, and though in rare cases, they can lead to serious or life-threatening events. The aim of this review is to evaluate the clinical meaningfulness of anti-β-amyloid therapies amidst amyloid-related imaging abnormalities concern in Alzheimer’s disease.

## Teaser

Anti-β-amyloid antibodies have demonstrated clinical efficacy but are associated with adverse events termed amyloid-related imaging abnormalities. Aljuhani *et al*. evaluate the potential clinical meaningfulness of the clinical trial results and safety concerns associated with anti-β-amyloid therapies and discuss the translation of efficacy into effectiveness in the real world.

## Graphical Abstract

**Graphical Abstract:**
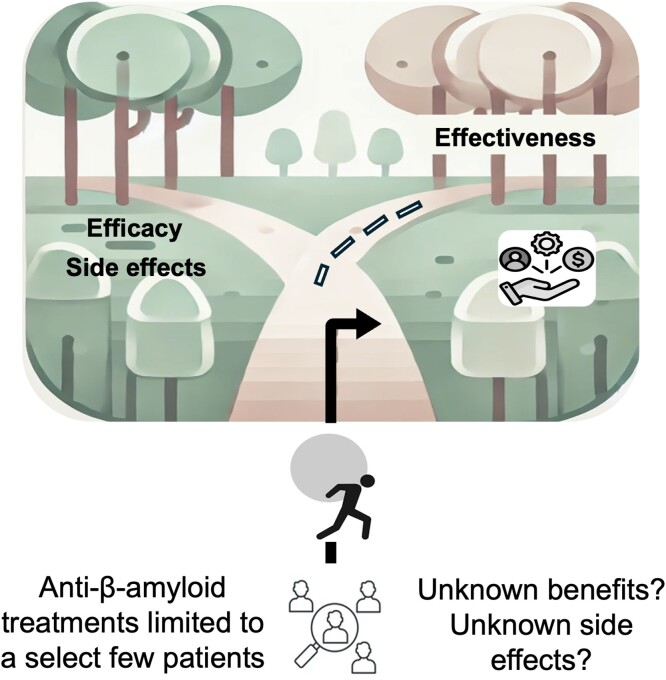

## Introduction

Alzheimer’s disease is the most prevalent form of dementia in the elderly, which is clinically characterized by a gradual and progressive deterioration of cognitive functions.^1^ Pathologically, Alzheimer’s disease is marked by synapse and neuron loss, β-amyloid (Aβ) plaque deposition, neuritic dystrophy, neurofibrillary tangles from hyperphosphorylated tau (p-tau), vascular changes and microglia- and astrocyte-driven inflammation.^2,3^ Aβ cascade hypothesis assumes a serial model of causality where Aβ triggers a cascade of events leading to NFTs, vascular events, inflammation, neurodegeneration and clinical dementia.^4^

Alzheimer’s disease begins with a prolonged preclinical stage, lasting 20–30 years, during which Aβ plaques accumulate without noticeable clinical symptoms.^5^ This is followed by a prodromal stage, typically lasting 3–5 years, characterized by mild cognitive impairment (MCI).^6-8^ Approximately 15% of individuals with MCI progress to dementia within 2 years,^9^ and around one-third develop Alzheimer’s disease-related dementia over 5 years.^10^ However, some MCI patients either remain stable or revert to normal cognitive function, with meta-analyses showing a reversion rate of 26%.^11^ A major focus of research is identifying which MCI patients are most likely to progress to dementia.^12^ Globally, it is estimated that 416 million people have preclinical Alzheimer’s disease, prodromal Alzheimer’s disease or Alzheimer’s disease-related dementia.^13^

Some MCI individuals do not experience further cognitive decline or revert to normal cognition. Population-based studies comprised of systematic review and meta-analysis found a reversion rate of 26%.^11^ Identifying which individuals with MCI may develop dementia is a key goal of current research.^12^ Worldwide, the estimated number of people with preclinical Alzheimer’s disease, prodromal Alzheimer’s disease and Alzheimer’s disease dementia is 416 million.^13^

**Figure 1:**
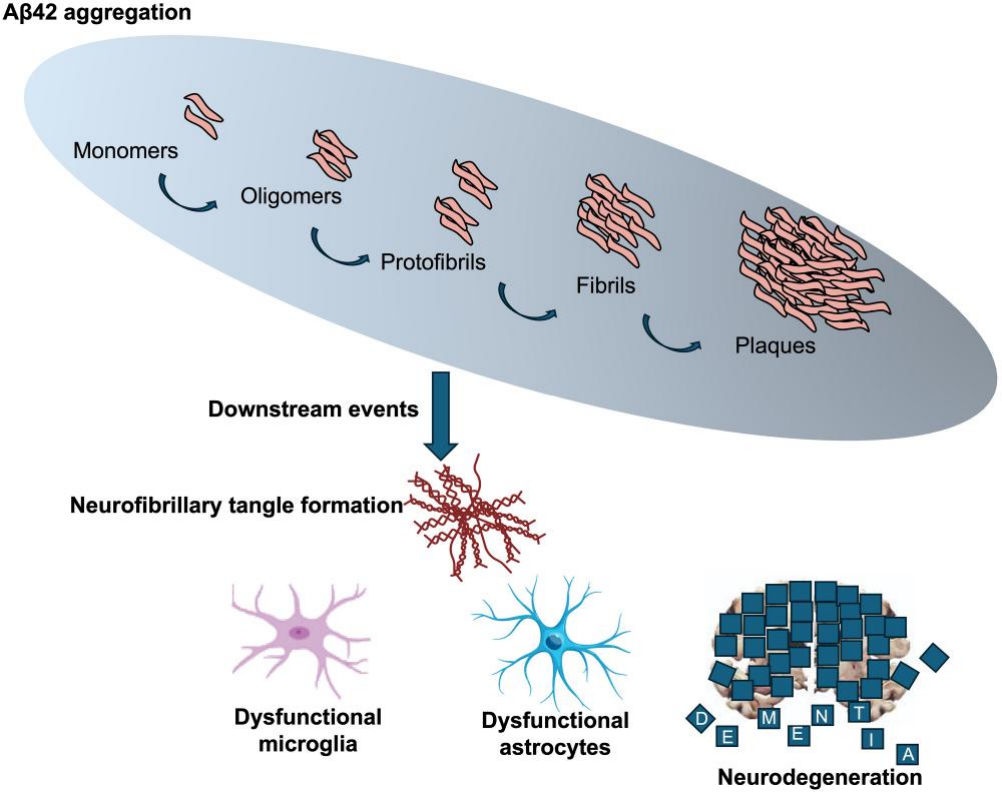
**Amyloid beta 42 aggregation drives neurodegeneration and pathology.** Schematic showing upstream amyloid beta 42 aggregation driving downstream pathology, which results in neurofibrillary tangles, glial dysfunction, neurodegeneration and clinical dementia. Aβ, β-amyloid.

The central and early role of Aβ in Alzheimer’s disease pathogenesis is supported by a plethora of studies including genetic analyses, biomarker research and genome-wide association studies in both familial (early-onset) and sporadic (late-onset) forms of Alzheimer’s disease (Fig. 1). The presence of Aβ species in the brain—soluble Aβ oligomers formed via aggregation of misfolded Aβ monomers—have consistently been correlated with acute neurotoxicity and neurodegeneration in Alzheimer’s disease. Preclinical and clinical evidence demonstrate that soluble Aβ oligomers, instead of insoluble aggregates (plaques and fibrils), propagate neurotoxicity and disease progression in Alzheimer’s disease.^14^ The upstream role of Aβ in driving the tau pathology and cognitive deterioration in patients with Alzheimer’s disease is reinforced by longitudinal Aβ and tau PET imaging studies. PET studies show that cortical Aβ burden needs to surpass a critical threshold prior to spreading of tau pathology from medial temporal lobes to the neocortex, accelerating cognitive deterioration.^15^ The sequence of pathologies suggests that targeting Aβ should ameliorate downstream tau pathology and cognitive decline, as observed in clinical trials involving aducanumab, lecanemab and donanemab.^16-19^

Monoclonal antibodies directed against Aβ demonstrate slowing of the clinical decline in early Alzheimer’s disease. Aducanumab, lecanemab and donanemab clinical trials showed slowing of Alzheimer’s disease progression on composite scores by 25–40% based on the measure used.^16-20^ Delaying clinical decline can be observed on cognitive scales, functional tools and primary composite outcomes. Lecanemab and donanemab have been granted standard approval by the US Food and Drug Administration (FDA). The clinical outcomes were supported by biomarker changes including marked Aβ plaque lowering demonstrated by Aβ-PET and effects on downstream biomarkers including p-tau 181, p-tau 217 and glial fibrillary acidic protein (GFAP).^16-18,21^ Monoclonal antibodies directed against Aβ species can cause side effects of bleeding and swelling in the brain, called amyloid-related imaging abnormalities (ARIA)—ARIA with oedema (ARIA-E) and ARIA with hemosiderin deposition (ARIA-H).^22,23^ Incidence and timing of ARIA vary among treatments. ARIA predominantly occurs early in treatment and is usually asymptomatic, but rare serious and fatal events, such as serious intracerebral haemorrhages >1 cm, have been reported in patients treated with this class of medications.^23^ Questions have been raised on the clinical meaningfulness of Aβ removal and the reported modest benefits, and whether the efficacy of the anti-Aβ treatments outweighs the risk of developing ARIA. What constitutes an abnormal amount (Aβ positive), and/or a pathological amount, of Aβ in the brain? What level and duration must anti-Aβ be administered for to achieve a therapeutic benefit that is clinically meaningful from the perspective of a clinician, patient and their care partner?^24,25^ The aim of this review is to evaluate the clinical meaningfulness of anti-Aβ therapies amidst ARIA concern in Alzheimer’s disease.

## Aβ oligomer toxicity remains a chimaera

Aβ neurotoxicity is dependent on the primary structure and aggregation state of Aβ. Two major forms of Aβ are produced in humans comprising of Aβ1–40 or Aβ1–42 amino acid residues. The relative proportion of Aβ1–42 is necessary for Alzheimer’s disease progression, as the longer form has greater propensity to aggregate and confers greater toxicity than Aβ1–40.^4^ Aβ molecules can form various species—low molecular weight oligomers, high molecular weight oligomers (protofibrils) and insoluble fibrils observed in plaques. Although Aβ aggregates may act directly on synapses to cause neuronal injury or indirectly through activating microglia and astrocytes, evidence favours the hypothesis that soluble oligomeric Aβ drives Alzheimer’s disease pathogenesis (i.e. the oligomer hypothesis).^4,26,27^ The scientific literature does not provide clarity on which specific Aβ oligomeric species are pathologically relevant in Alzheimer’s disease. The multiplicity of Aβ species is a result of native biological variation as well as technical variability arising due to lack of standardized tools to measure Aβ species. The fundamental question on the role of oligomeric Aβ species in cognitive impairment and neuronal death in Alzheimer’s disease dementia remains.^28^

Nonetheless, removal of soluble Aβ aggregates is the approach adopted in recent clinical trials of Alzheimer’s disease.^16-19^ Lecanemab preferentially targets Aβ protofibrils and has been shown to inhibit vascular and inflammatory activation. Lecanemab blocks the binding of coagulation factor XII and high molecular weight kininogen to Aβ and prevents Aβ protofibril-induced acceleration intrinsic coagulation in human plasma.^29^ Lecanemab causes lower rates of ARIA compared with other anti-Aβ antibodies such as aducanumab, gantenerumab, or donanemab.^16-19^ The effective inhibition of the vascular and inflammatory activation by lecanemab may reduce bradykinin production and therefore reduce the occurrence of ARIA in patients.

## Binding profiles of anti-Aβ antibodies might explain efficacy and side effects

**Figure 2:**
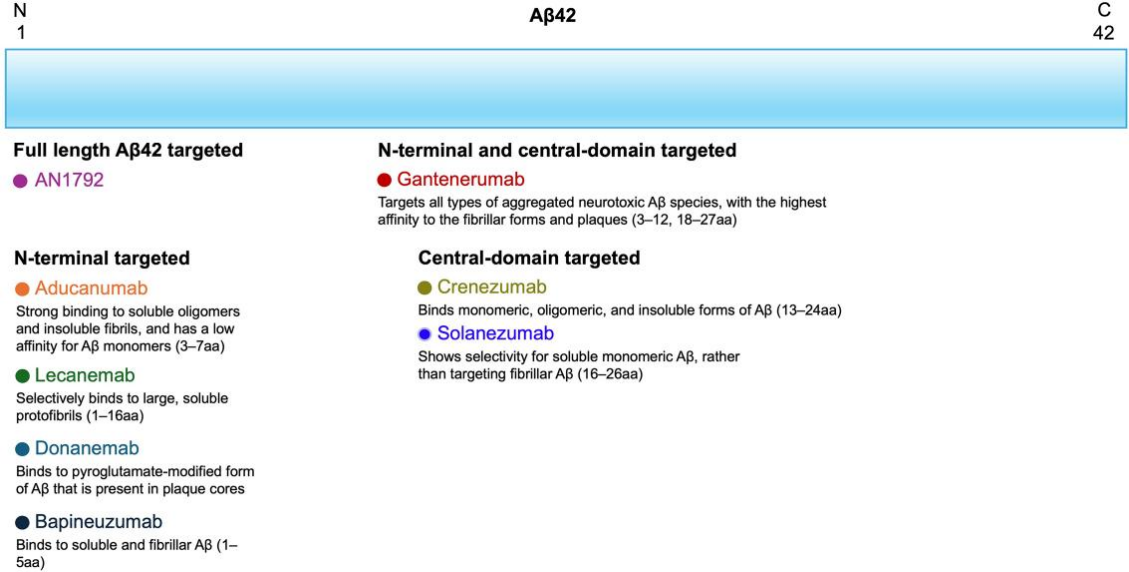
**Anti-Aβ monoclonal antibodies bind to different species of Aβ42.** aa, epitope refers to the location of the targeted amino acid from the N-terminal of the Aβ peptide segment; Aβ, β-amyloid; C, C-terminus; N, N-terminus.

**Figure 3:**
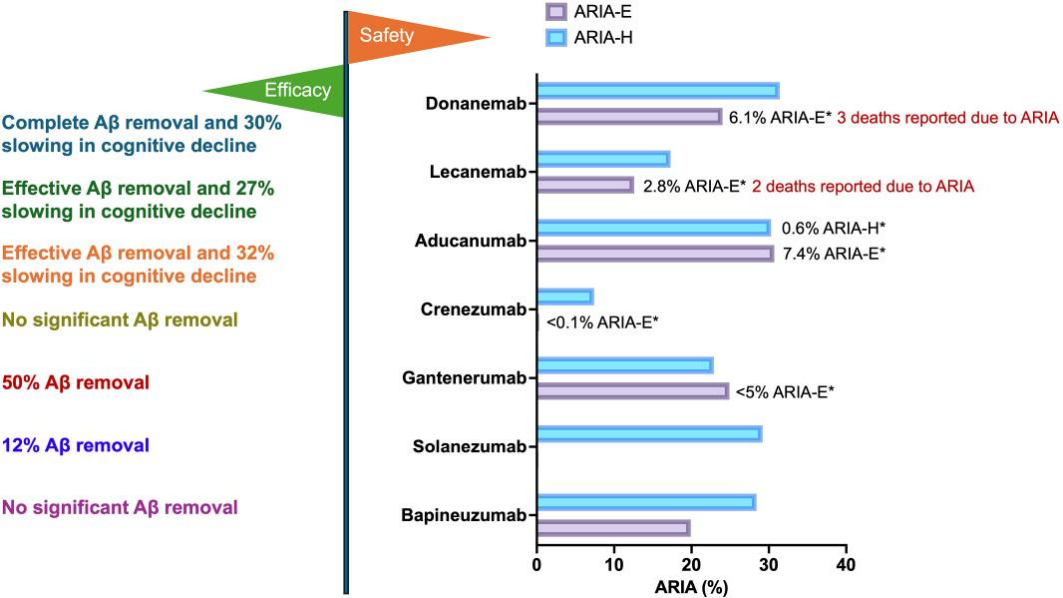
**Efficacy and safety of anti-Aβ monoclonal antibodies in phase 3 clinical trials in Alzheimer’s disease.** Phase 3 clinical trials included bapineuzumab (*N* = 2452), solanezumab (1169), gantenerumab (1965), crenezumab (1619), aducanumab (3285), lecanemab (1795) and donanemab (1182). The bars in the plot show the percentage of ARIA-E and ARIA-H. The annotations shown next to the bars in the plot show the percentage of symptomatic ARIA-E and ARIA-H if reported in the clinical trials, marked with an asterisk. *Symptomatic adverse events. Aβ, β-amyloid; ARIA, amyloid-related imaging abnormalities; ARIA-E, ARIA-oedema; ARIA-H, ARIA-haemorrhage.

The human brain is composed of intricate mixtures of different Aβ species of varied sizes, which includes N- and C-terminal truncations, and posttranslational modifications. Anti-Aβ antibody binding profiles to different Aβ species and the specific terminus may explain the efficacy and side effects in Alzheimer’s disease (Figs 2 and 3).^16,18,20,24,30^

There were 1653 and 1638 people with early Alzheimer’s disease [MCI attributable to Alzheimer’s disease (80%) or moderate Alzheimer’s disease (20%)] in two identically planned 18-month randomized, double-blind, placebo-controlled, parallel-group investigations (ENGAGE and EMERGE), respectively.^31^ Patients were randomly assigned (in a 1:1:1 ratio) to receive placebo or low-dose aducanumab (3 mg/kg for carrier of APOE-ε4 allele and 6 mg/kg otherwise) and high-dose aducanumab (6 mg/kg for carrier of APOE-ε4 allele and 10 mg/kg otherwise). The drug–placebo difference on the CDR-SB score at 78 weeks was selected as the primary outcome measure. After safety of the lower doses were established in trials, 10 mg/kg became an allowable dose for APOE ε4 carriers. The ENGAGE and EMERGE studies were both halted after a futility analysis of data from the first 50% enrolled participants. Phase 3 outcomes were further evaluated using larger and more extensive data sets through prespecified statistical analyses. The EMERGE programme demonstrated that patients receiving high-dose aducanumab arm experienced a significant reduction in progression of disease specifically on the CDR-SB scale meeting its primary end-point (22% decrease, *P* = 0.012) at Week 78 following exploratory analysis.^16^ The high-dose arm of this trial also successfully met its secondary end-points—MMSE, ADAS-Cog 13 and AADCS-ADL-MCI. On the other hand, ENGAGE trial was unsuccessful at attaining the primary and secondary end-points. Sub-analyses of ENGAGE and EMERGE results exhibited dose- and time-dependent decreases in PET Aβ with aducanumab treatment. Dose-related decreases were demonstrated in downstream Alzheimer’s disease biomarkers: tau PET, CSF p-tau and plasma p-tau 181.^16^ These data demonstrate that removing Aβ ameliorates the downstream tau pathology and cognition. In the high-dose arms, ARIA-E accounted for 35% of adverse events, whereas ARIA-H accounted for 19.1%.

Long-term analyses of patients receiving 4 years of aducanumab demonstrated a dose- and time-dependent reduction in Aβ PET and clinical benefits on the CDR-SB and MMSE.^31^ Aducanumab was the first anti-Aβ therapy to be approved by the FDA in 2021 but will be discontinued by its manufacturer (Biogen) in 2024 (not for reasons related to efficacy or safety but to refocus resources in Alzheimer’s disease).

Gantenerumab did not lower Aβ below a level presumably necessary to slow Alzheimer’s disease progression and provide one with clinical benefit.^31^ TRAILBLAZER-ALZ 2 is a phase III study of donanemab that enrolled 1800 participants with early symptomatic Alzheimer’s disease. Donanemab slowed the rate of clinical decline by 35% in CDR-SB and by 40% in iADRS for participants with intermediate levels (*n* = 1182) at baseline who also had symptomatic disease similar to those evaluated in EMERGE. In the donanemab group, ARIA-E occurred in 24% of patients; symptomatic ARIA-E affected 6.1%. In participants who received the donanemab, 31% had ARIA-H events compared with 13% receiving placebo.^20^ Donanemab has received standard FDA approval based on the results.^31,32^

Lecanemab binds to the protofibrils rather than Aβ plaque, a preference ratio of 10:1 between lecanemab binding at plaques compared with plasma levels and an affinity that is >100-fold higher for this target over Aβ monomers. Donanemab targets plaque-specific pyroglutamate Aβ. The proposed mechanism for all mAbs is reduction of Aβ plaques through microglial-mediated phagocytosis and subsequent fibrillar Aß degradation in the endosomal/lysosomal system. The lecanemab clinical trial ‘CLARITY AD’ was conducted in 1795 participants randomized at a 1:1 ratio to receive either biweekly dosing of lecanemab, every 4 weeks (10 mg/kg) or placebo. Eligibility was based on age (50–90 years), MCI or mild Alzheimer’s disease diagnosis (National Institute on Aging–Alzheimer’s Association criteria), 1 SD below the age-adjusted mean in objective episodic memory performance, as measured with WMS-IV LMII, and Aβ positivity defined by PET imaging or CSF measurement of Aβ1–42. CLARITY AD enrolled more patients from racial and ethnic minority communities than previous trails. The global enrolment of a diverse group of participants (20% non-White) was further enriched by inclusion from the USA, with 6.1 and 28.1% Black and Hispanic individuals among those screened for eligibility, respectively; Black and Hispanic participants comprised 4.5 and 22.5% randomly assigned to treatment versus standard-of-care tailoring arms). The modified intention-to-treat primary end-point of the CLARITY AD study was changed from baseline in the CDR-SB at 18 months. Lecanemab reduced clinical decline on the CDR-SB by 27% over 18 months, corresponding to a −0.45-point change (+1.21 versus +1.66) compared with placebo. Scores for other cognitive measures with lecanemab versus placebo (ADAS-Cog, ADCOMS and as well-revised BEHAVE-ADFMS) were significantly delayed at 18 months. The Aβ PET plaque levels were decreased on lecanemab (−55.48 centiloid change) compared with placebo (+3.64 centiloid change). However, NfL is less tissue specific to neurodegeneration than the other markers and has a slower change in response compared with Aβ, tau or GFAP (neuroinflammation).^18,31^

A total of 26.4% of patients in the lecanemab arm experienced infusion-related reactions, while 17.3% experienced ARIA-H and 12.6% experienced ARIA-E. In the lecanemab arm among non-carriers of APOE ε4, fewer individuals experienced ARIA-H (11.9%) and ARIA-E (5.4%); a higher number of APOE ε4 heterozygotes experienced ARIA-H (14%) and ARIA-E (10.9%); and APOE ε4 homozygotes had the highest incidence of ARIA-H (occurring in 39% of patients) and ARIA-E (occurring in 32.6%).^18^ The FDA has given lecanemab traditional approval based on the data from CLARITY AD. Lecanemab is also being tested in two other clinical trials, a phase 3 trial (AHEAD 3-45) involving CN individuals with high brain Aβ levels and the first DIAN-TU prevention study to combine an early anti-Aβ therapy with simultaneous tau targeting therapies in patients with familial Alzheimer’s disease mutations—pairing lecanemab with Eisai’s humanized mAb E2814.^33^ A subcutaneous form of lecanemab is under consideration and may offer flexibility of treating patients at home obviating the need for saturated infusion centres.

The reason why lecanemab and donanemab have worked is due to the rate and degree of Aβ removal occurring over a sufficiently long period resulting in clinical benefits.^24^ Lecanemab and donanemab effectively removed Aβ^18,20^; EMERGE (aducanumab) that showed Aβ removal over enough time demonstrated clinical benefit; whereas ENGAGE (aducanumab) that showed less Aβ removal did not produce clinical benefit.^16^ It will be interesting to evaluate the epitope binding properties of anti-Aβ antibodies to understand their efficacy and side effect profiles.^30^ The crucial aspects to understand are the pathogenic underpinnings of the remaining cognitive decline when Aβ is cleared, and whether this residual decline is entirely Aβ independent. If this is the case, the work is cut-out to determine the substrates (e.g. NFTs) underpinning the residual cognitive decline. If Aβ is cleared prior to the development of the disease or early in the disease for a long period of time, will this prevent and/or lead to (persistent) cognitive improvement?

## Defining clinical meaningfulness with anti-Aβ treatments

A major obstacle is being able to quantify the clinical significance of Alzheimer’s disease treatments. The minimal clinically important difference (MCID) is the smallest change in score in the domain of interest that patients perceive as beneficial and which would mandate, assuming experienced no troublesome side effect or excessive cost, a change in patient management.^34^ Meaningful clinical benefits of anti-Aβ treatments are of great value to stakeholders—patients, care partners and healthcare decision makers including physicians. To our knowledge, empirical information on MCID estimates across a variety of clinical trial outcome assessments to quantify cognitive change in dementia has not been systematically collected.^35,36^ For the other problem in dementia, clinical significance was reported for just under half of 57 trials (46%).^35^ There is no standardized approach for estimating MCID. There are two most frequently mechanisms used to deﬁne MCID, namely the anchor-based approach and distribution-based approach.^37,38^ An anchor-based approach refers to a meaningful change in an outcome measure relative to some external anchor, such as the patient’s perception or clinical opinion. The distribution-based approach is applicable to clinical trial populations only, because it calibrates MCID from variation observed among patients in the trials. Among trials of dementia, estimates for MCID have shown substantial variation. A survey of neurologists and geriatricians reported a mean MCID for MMSE of 3.75 while the DOMINO trial estimated MMSE to be 1.4 points.^39,40^ Another challenge confronted by Alzheimer’s disease is that the MCID will likely differ across the disease continuum because calibration of estimates between stages of cognitive impairment may vary.^41^

Clinicians use their evaluation of significant declines in patients’ cognitive, functional or behavioural characteristics since the last visit as a reference point. Over the course of a year, an average increase of 1–2 points in the CDR-SB, a decrease of 1–3 points in the MMSE and an increase of 3–5 points in the FAQ are regarded as clinically significant changes. This begins to challenge the notion that thresholds for clinically meaningful decline increase from MCI–AD to moderate–severe Alzheimer’s disease on the disease severity spectrum.^38^ But estimates of what constitutes a meaningful change on the basis only of baseline score distribution did not vary much and overall supported findings from anchor-based questioning. These identify known MCID in disease severity that could be used to calibrate the MCID on similar scales for use in tests of treatments. The results should be interpreted with caution: The National Alzheimer’s Coordinating Centre database, from which the data were derived, may not be representative of all patients diagnosed with Alzheimer’s disease because patient characteristics and enrolment practices vary. Additionally, the diagnostic criteria for MCI due to Alzheimer’s disease were not consistent with current clinical practice and few subjects underwent biomarker verification. Meaningful changes were ascertained by a binary anchor to patients, and what proportion of this is tantamount to the ‘minimum’ threshold remains unclear. The study was based on clinician opinion alone, without perceptions from the patient or care partner subject to disease variation.^42^

Available evidence indicates that an end-of-trial Aβ level >25 centiloids posits a lack of slowing of disease progression, regardless of extent of total reduction in Aβ load. Such patients may experience the greatest reductions if they start with high baseline Aβ levels compared with a patient whose end-of-trial Aβ is >25 centiloids and who shows no benefit.^31^ Sufficient levels of Aβ reduction were achieved in the positive trials [lecanemab, donanemab and aducanumab (EMERGE)], but were not achieved in the negative gantenerumab and aducanumab (ENGAGE) trials.^16-19,43^ Because the threshold of Aβ reduction needed to delay disease progression must be dose related, trials using higher doses may reach this necessary level of reduced Aβ load. Studies should aim to uncover the appropriate weight-adjusted dosing strategy for obtaining brain Aβ exposures within specified dose ranges across a continuum of body weights, which will inform patient selection and prescription practices in anti-Aβ therapies.^31^ It appears that a certain threshold needs to be met to alter Alzheimer’s disease biology to translate into clinical efficacy. A critical range of 15–25 centiloids is considered of importance in natural history studies of Alzheimer’s disease.^44,45^ If patients who had a negative Aβ PET at baseline reached an inflection point of 15–18.5 centiloids, this was predictive of future pathological Aβ accrual and decline on the Preclinical Alzheimer’s Cognitive Composite.^46^ A threshold of 25 centiloids coincides with the peak rate of Aβ accumulation and is correlated with Aβ and tau biology.^45^ Aβ levels of 25 centiloids is associated with increasing p-tau 231 and p-tau 217 levels to 2 SDs above normal. Increased tau PET SUVR occurs once Aβ levels surpass the 25 centiloids landmark.^47^ Aβ levels ≥25 centiloids coincide with cognitive impairment, while cognitive benefit is apparent between 15 and 25 centiloids, and an association with tau biology may instigate accrual of pathological tau biomarkers.^31^

In the phase 3 clinical trial, lecanemab lowered CDR-SB by a difference of 0.45 compared with placebo, which corresponds to a delay in cognitive decline on the CDR-SB by 27%. The insidious nature of Alzheimer’s disease progression renders a 27% slowing in decline difficult to detect among key stakeholders such as clinicians, patients, caregivers and payers.

The clinical meaningfulness of the degree of slowing of clinical decline is contentious. On clinical and functional measures, the drug–placebo difference is ∼30%. According to the donanemab phase 2 trial results, this drug–placebo difference represents about a 5-month delay in cognitive decline over an 18-month study.^48^ In the phase 3 lecanemab trial, a 0.45 decline in CDR-SB was observed with lecanemab compared with placebo, indicative of a slowed clinical progression by 27%.^18^ Due to the insidious nature of Alzheimer’s disease progression, a 27% slowing in decline is challenging to perceive for stakeholders including clinicians, patients and their care partners, policymakers, payers and regulatory bodies. Simulation modelling estimated that lecanemab will delay the mild Alzheimer’s disease phases of dementia by an additional 2.5 years with net cost savings.^49,50^ Only a 25% reduction is often used as a cut-off point for clinical significance.^17,51^ For example, if a 25% slowing of progression in the treatment group compared with placebo is assumed, this will be reflected as an absolute reduction of decline by one-half point for those on anti-Aβ versus placebo, indicative of a 3-month delay in decline for the treatment group (suggestive of a 3-month slowing of disease progression). The same half point difference translates to a delayed decline of 6 months at 18 months between treated and control groups, and a difference of 7.5 months at 24 months. During 24 months in early-stage Alzheimer’s disease, a deceleration of decline on this scale would be interpretable and clinically meaningful to patients, care partners and clinicians as representing >6-month delay in disease-related clinical progression.^52^

## Managing the E’s and H’s in ARIA

Pooled data from multiple randomized trials show that each 0.1-unit reduction in PET Aβ SUVR is correlated with a reduction (95% confidence interval) by 0.09 (0.034–0.15) point in the average change for CDR-SB, 0.33 (0.12–0.55) for ADAS-CS and 0.13 (0.017–0.24) for MMSE.^53^ Results suggest that Aβ plaque is an amenable biological target for modifying Alzheimer’s disease pathophysiology. These results suggest that there might be considerable heterogeneity in the causal relationship of Aβ clearance processes with cognitive and functional trajectory, which deserves further examination to characterize it as comprehensively and accurately as possible.

Since lecanemab is FDA approved, ∼2000–3000 people across the US dementia clinics are on lecanemab treatment. This is predominantly happening at the larger specialized neurology centres (e.g. Columbia University, Emory University in Atlanta and the Mayo Clinic in Rochester, MN) with clinical trial experience of using anti-Aβ therapies (Alzforum news, 26 January 2024). Although the rollout has been smooth, with an emphasis on safety, the next hurdle will be to rollout lecanemab treatment at less-experienced clinics, as the treatment bottleneck is beginning to tighten. It is estimated that 17% of the 1–2 million US individuals with early Alzheimer’s disease will meet the lecanemab phase 3 inclusion and exclusion criteria.^54^ This will amount to ∼170 000–340 000 individuals requiring treatment with lecanemab, a 100-fold increase from the current number being treated. There are 550 certified cognitive neurologists in the USA, which will mean that there will be one cognitive neurologist for every 600 patients (Alzforum news, 26 January 2024).

To cope with existing and future challenges, Centre of Excellence should be established and affiliated with the less-experienced centres to formulate an effective dementia ecosystem. Emphasis should be placed on engagement of multidisciplinary teams for improved management of ARIA. Ongoing education on appropriate use guidelines for lecanemab (and donanemab) to ensure effective management strategies on ARIA will be required.^22^ The use of lecanemab (and donanemab) may be avoided in patients on anticoagulant or who have pre-existing cerebral amyloid angiopathy. A cautious approach is warranted in patients homozygous for APOE ε4. Further evidence is required to better understand the association between APOE ε4 polymorphisms and ARIA events. Patient, families and care partners should be educated on the benefits and risks of treatments, provided options for symptom management or pre-treatment if required. The chronic effects of ARIA and brain atrophy on disease progression are far from understood, given that trial data and MCID estimates may be challenging to generalize to clinical populations. Documentation of patients on lecanemab (and donanemab) in the real-world space through the CMS registry will help to inform on the optimal clinical decision-making. The chronic effects of ARIA and brain atrophy along with their clinical repercussions should be monitored by the funding bodies and institutions. To evaluate safety measures concerning anti-Aβ clinical trials, details of radiological severity, clinical severity and clinical outcomes associated with ARIA should be disclosed and published in peer-reviewed journals. Sponsoring bodies should in a timely manner implement a data-sharing plan to make individual patient-level clinical trial data publicly accessible enabling external validation of study design and data analyses. Open-label extension studies should provide more regular updates of safety data, with companies periodically encouraged to share open access clinical trial findings. The deficiencies in risk analysis and mitigation strategy development suggest that new studies reporting both trial data and MCID benchmarks specific to patients with Alzheimer’s disease seen in clinical practice are urgently needed. Alternative trial designs should be considered to study whether clinically relevant within-patient treatment effects or disease modification has occurred, such as delayed-start and staggered-withdrawal designs. The availability of potential disease-modifying pharmacologic treatments for Alzheimer’s disease represents an exciting and awaited landmark not only in the history of Alzheimer’s disease but also its clinical management. Outstanding questions remain due to limited trial data available from clinical trials of anti-Aβ compounds, with potential for biases introduced by unblinding and dropout at different times related to the safety question as well as whether any such agent is recognized clinically or in a cost-effective manner. These outstanding issues should be focussed on by regulators and payors when deciding on drug approvals, and by clinicians and patients (and care partners) once treatments are licenced, to carefully evaluate the evidence and make an informed decision.^55^

**Figure 4:**
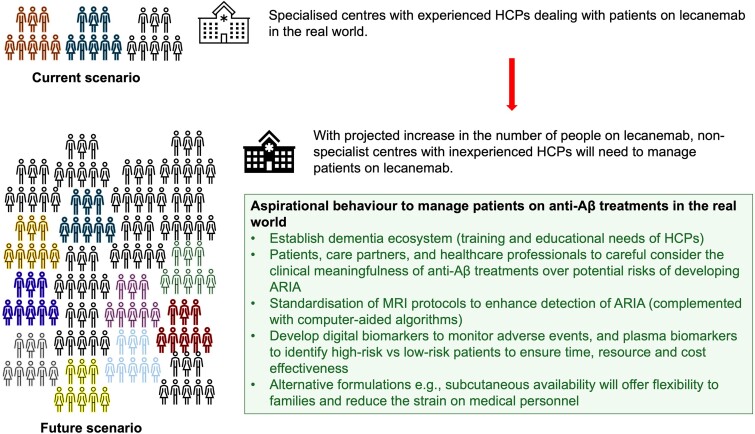
**Approaches to address increasing use of anti-Aβ treatments**. Strategies to cope with the projected rise in the number of people likely to be administered anti-Aβ treatments. Aβ, β-amyloid; ARIA, amyloid-related imaging abnormalities; HCPs, healthcare professionals.

Future availability on flexible options, i.e. subcutaneous formulations of anti-Aβ treatments, will ease the strain on clinics and increase convenience for patients, families and care partners. Digital biomarkers and plasma biomarkers may help to stratify patient’s risk and inform on who requires an immediate and/or follow-up MRI to assess for ARIA, which will have financial, societal and logistical implications (Fig. 4). The availability of standardized and well-defined clinical monitoring and management protocols are needed to adequately identify ARIA in real-world settings and monitor its progression over time.^23^

Exaggerated brain atrophy in the form of enlarged ventricular CSF volume or hippocampal/whole brain shrinkage has been observed for anti-Aβ therapies including lecanemab, donanemab and aducanumab.^16,19,56^ This is worrying as accelerated brain volume alterations in Alzheimer’s disease have been associated with enhanced neurodegeneration, particularly outside clinical trial settings. ARIA is considered a putative cause of altered brain volume and warrants extensive investigation. A meta-analysis showed a correlation between ARIA frequency and enlarged ventricular volume, which was associated with exposure to anti-Aβ antibodies. Further, among MCI patients treated with anti-Aβ drugs, a substantial proportion was estimated to decline in brain volume back into the range typical of Alzheimer’s disease ∼8 months earlier than they would have without treatment.^57^

In donanemab-treated patients, it was suggested that reduction in the Aβ plaque volume accounted for the accelerated brain volume loss, compared with the placebo-treated group.^17^ Between treatment arms, the total Aβ load is ∼6.5 mg and difference in brain volume (placebo versus donanemab) is ∼5 mL.^58^ Assuming Aβ and volume, that is being generous since donanemab removes all the brain Aβ and protein density = 1.35 g/mL, then ∼Aβ should only occupy 0.0048 mL. These assumptions are exaggerated; however, this is still <1000 times bigger than the reported difference in brain volume.^56^

While reducing Aβ has shown cognitive benefit, it may be neutralized over time on the occurrence of secondary inflammation. Anti-Aβ antibodies cause ARIA on removal of vascular Aβ. As such, MRI may be unable to detect inflammation due to brain parenchymal Aβ removal, which may be detrimental. In the donanemab clinical trial, there was an 80% reduction in Aβ plaque load by 6 months while the brain atrophy was delayed by >12 months.^17^ This suggests that reduced Aβ plaque deposition cannot explain the brain volume loss. Since the brain atrophy is delayed despite substantial reduction in Aβ plaque load, longer clinical trials are warranted to monitor cognitive deterioration due to anti-Aβ antibody-induced brain damage.^56^ The brain volume loss is considered a proxy of brain tissue loss and likely a proximate cause of cognitive dysfunction and indicative of disease progression in Alzheimer’s disease.^59,60^

Neuropathological examination of patients who had reportedly died from active ARIA had vascular inflammation throughout the brain. The pathophysiology of ARIA is like CAA and may have a mechanistic connection with inflammation in the context of CAA. In contrast, Aβ-immune complexes activate perivascular macrophages to cause blood vessel injury. For instance, an MRI of a 79-year-old woman with Alzheimer’s disease who had been in the CLARITY AD lecanemab (phase 3) trial showed multifocal hyperintensity and dramatic new development of cerebral microhaemorrhages. She received antiepileptic treatment and high-dose i.v. corticosteroids, yet her condition deteriorated after 5 days to the point of death post-mortem MRI confirmed severe microhaemorrhages in the temporal, parietal and occipital cortices. Post-mortem examination revealed homozygosity for APOE ε4 and neuropathological features of intermediate severity Alzheimer’s disease (neuritic plaques, Braak neurofibrillary tangles V/VI) with severe CAA accompanied by perivascular lymphocytic infiltrates, abundant reactive macrophages and fibrinoid degeneration of vessel walls. There were numerous microaneurysms with deposits of Aβ in meningeal vessels and penetrating arterioles. The patient probably succumbed because of catastrophic cerebral amyloid-related inflammation.^61^ Patient’s MRI at time of death was compared with baseline (prior to the open-label extension). At baseline, there were four microhaemorrhages below the treatment threshold for lecanemab. The baseline MRI was suggestive of probable CAA, but this did not meet exclusion criteria in the CLARITY trial. FDA had initially suggested excluded individuals with ≥2 microhaemorrhages from participating in anti-Aβ trials. The standard recommendation of 4 was devised by the working group convened by the Alzheimer’s Association.^6^ Presence of ≥2 microhaemorrhages at baseline doubled a person’s risk of developing ARIA-E. Based on preclinical evidence, it appears that antibodies interact with vascular Aβ to activate perivascular (and leptomeningeal) macrophages,^62^ which may be associated with ARIA. Similarly, post-mortem evaluations from cases treated with AN1792 and bapineuzumab demonstrated worsening of CAA.^63,64^ The underlying mechanism may involve breaking of Aβ oligomers from plaques, and its drainage into the basement membrane surrounding the blood vessels, which causes inflammation and vascular damage.

Standardization of MRI protocols to enhance detection of microhaemorrhages and probable CAA is warranted. A minimum field strength of 3 T using susceptibility-weighted imaging at a slice thickness ≤5 mm, is suggested to detect brain bleeds at a higher resolution.^61^ This presents a challenge as multiple hospitals and outpatient centres will require upgrades from 1.5 to 3 T scanners. The location of brain bleeds may provide insights into underlying pathophysiology; for example, lobar bleeds may be suggestive of extensive vascular Aβ. More aggressive immunosuppression can be considered for treatment of severe ARIA.^65^ Moreover, in autoimmune types of vasculopathy, interleukin (IL)-6 drives inflammatory response in both the vessel walls and the systemic circulation.^66^ Individuals with increased inflammatory markers (C-reactive protein) or erythrocyte sedimentation rate are at an elevated risk for CAA-related inflammation.^67^ It would be interesting to explore the role of IL-6 in the complications of CAA-related inflammation and ARIA-E, and whether repurposing of existing drugs such as tocilizumab (anti-IL-6 receptor used in giant cell arteritis^68^) and siltuximab (anti-IL-6 used in idiopathic multicentric Castleman disease^69^) will lower risk of ARIA events.

## Is Aβ PET imaging the right tool in the management of Alzheimer’s disease?

With the imminent possibility of expansion of anti-Aβ antibodies, the use of Aβ PET imaging as a single primary surrogate efficacy measure in Alzheimer’s disease (as occurred during the accelerated approval of aducanumab^70^) is controversial and challenging to justify.^71,72^ Firstly, the number of brain Aβ plaques measured with Aβ PET imaging does not correlate with the severity of cognitive impairment. There is no description of an Aβ plaque–only dementia, while tau-only pathology/tauopathy has been shown to cause neuronal loss in frontotemporal lobar dementia, and abnormalities occurring primarily in tau metabolism can lead to other dementia types including dementia pugilistica. This is in keeping with the amyloid cascade hypothesis, where Aβ acts as the trigger activating a cascade of events leading onto tau hyperphosphorylation, and neuronal death. It can be hypothesized that the onset of cognitive decline in Alzheimer’s disease happens when tau-mediated neuronal loss and dysfunction overwhelm the brain cognitive reserve. The modest clinical improvement apparent in anti-Aβ therapies suggests that removal of Aβ may experience a bottleneck, i.e. there may be a limit to the reduction in cognitive decline with anti-Aβ therapies. There may be more merit in utilizing anti-Aβ therapies in preventing accumulation of Aβ and thereby disease prevention in patients with Down’s syndrome, Alzheimer’s disease mutation carriers and perhaps early cases of Alzheimer’s disease. The current scenario where anti-Aβ therapies offer a modest benefit against the serious consequences of ARIA, which will be challenging to monitor in the real world, necessitates the need for transparency in reporting all the clinical trial results, including brain volume reductions associated with anti-Aβ treatments. The use of Aβ PET scans will act as a measure of target engagement (for anti-Aβ treatments) rather than that of clinical efficacy and may not be the most suitable entity to measure for monitoring disease progression.^24,28^

Secondly, ARIA resulting from anti-Aβ antibodies may locally affect blood–brain barrier diffusion and decrease tissue accumulation of PET biomarkers, irrespective of Aβ deposition.^73^ Attributing lower brain Aβ PET signals in patients with ARIAs in its entirety to anti-Aβ treatments warrants thorough scientific scrutiny. Moreover, large non-specific white matter signals observed with Aβ PET imaging tracers including ^18^F-florbetapir results in significant spill-over and partial volume effects over the cortical signals.^74^ This challenges the grey matter signal quantification in the presence of cortical atrophy observed in Alzheimer’s disease brain.^71^

^18^F-FDG-PET global quantification is considered a superior indicator of cognitive performance in patients with MCI and Alzheimer’s disease compared with ^18^F-florbetapir PET.^72,75^ The *in vivo* pattern of regional cerebral glucose hypometabolism (measured by ^18^F-FDG-PET) is observed in most clinically diagnosed patients with Alzheimer’s disease and in >85% of pathologically confirmed Alzheimer’s disease cases.^76^ Glucose hypometabolism closely coincides with the extent/severity of cognitive impairment in dementia.^77,78^ In contrast, a significant number of patients clinically diagnosed with Alzheimer’s disease do not have high levels of Aβ in their brain. The correlation between Aβ PET and cognition is weak, and the distribution of Aβ plaques does not correlate with clinical symptoms in Alzheimer’s disease.^79,80^ Longitudinal studies have found a lack of progression in Aβ PET uptake in cognitively normal, MCI and Alzheimer’s disease, with the rate of change not differing between clinical groups.^81-83^ Alzheimer’s disease patients apparently reach a plateau in brain Aβ PET retention, despite progression of their clinical symptoms and worsening of hypometabolism on ^18^F-FDG-PET.^81^ Based on the evidence discussed, ^18^F-FDG-PET should be recommended over Aβ PET in the evaluation for MCI and Alzheimer’s disease.^75,84^ It is likely that baseline and follow-up ^18^F-FDG-PET scans will be required for demonstrating significant side effects associated with anti-Aβ treatments, and as such prospective studies should be conducted to more fully determine the benefit versus risk ratio of anti-Aβ treatments.

## Public health perspective

Short-term reductions with anti-Aβ treatments in cognitive decline are small, with frequent adverse events that have uncertain outcomes and unknown long-term effects. The treatment regimens appear to place huge burden on patients and their care partners. It is essential to provide balanced information to patients and their care partners, and clinicians to aid decision-making. This includes considerations about the potential alterations to patients’ existing treatment regimens (and its impact) to meet the eligibility criteria, for example, stopping anticoagulants to limit bleeding risk related to anti-Aβ treatments.^85^

The clinical trials included patients with early symptomatic Alzheimer’s disease and excluded patients with co-neuropathologies that may contribute to their symptoms. The mismatch between trial cohorts, relatively young with less co-pathologies and co-morbidities, and real-world Alzheimer’s disease populations has profound implications for what effects may be observed once the anti-Aβ treatments are widely rolled out. It is not uncommon to find patients with a high prevalence of mixed dementia pathology and co-morbidities in the real world. An analysis of the National Alzheimer Coordinating Centre database (USA) showed that 20% of patients were diagnosed with mixed dementia, 21% had clinical depression, and 5% had a history of stroke.^86^ Alzheimer’s disease populations in the real world are more complex than the highly selective participants enrolled in clinical trials, which will likely result in dilution of a trial efficacy to below the estimated thresholds of MICD with potentially high rate of side effects (ARIA of unknown prognostic implications), which is unlikely to lead to population benefit. This results in restriction of treatments to narrowly defined patient populations. A population-based Mayo Clinic Study of Aging in the USA showed that only 8% of those with MCI or mild dementia with increased Aβ levels met the eligibility criteria from the lecanemab trial.^54^ Even if anti-Aβ treatments are approved for a small proportion of patients with early Alzheimer’s disease, it comes with considerable resource requirements and costs.

Unlike its FDA counterpart, the European Medicines Agency (EMA) has denied marketing authorization for lecanemab as it does not believe that the benefits outweigh the risks (Table 1). The EMA is reassessing its rejection following appeal by Biogen/Eisai. The UK’s Medicines and Healthcare Products Regulatory Agency (MHRA) has approved lecanemab for use in the UK to treat early-stage Alzheimer’s disease. However, UK’s National Institute for Health and Care Excellence (NICE), which makes evidence-based value-for-money judgements for the tax-funded healthcare system, has not recommended lecanemab to be made available on the NHS as benefits do not justify the high cost and resources required. NICE’s evaluation criteria include mortality, ability to remain independent and admission to full-time care, for which the evidence is sparse. Assessment of long-term clinical outcomes is dependent on the predictive value of Aβ removal as a surrogate end-point, but NICE and EMA do not support this position. Decisions from EMA and MHRA on donanemab are expected later this year. The current scenario-based analyses suggest that it will be a challenge for anti-Aβ treatments to significantly reduce population-level dementia morbidity at scale.^85^

**Table 1:** Approval of anti-β-amyloid therapies worldwide

	Anti-β-amyloid therapies		
Regulatory bodies	Aducanumab	Lecanemab	Donanemab
Food and Drug Administration (USA)	✔	✔	✔
European Medicines Agency (Europe)	✖	✖	
Medicines and Healthcare products Regulatory Agency (UK)	✖	✔	
Ministry of Heath, Labour and Welfare (Japan)	✖	✔	✔
National Medical Products Administration (China)		✔	
Department of Health (Hong Kong)		✔	
Ministry of Food and Drug Safety (South Korea)		✔	
Ministry of Health and Prevention (UAE)	✔	✔	

✔, approval; ✖, rejection. No symbol means the drug has either not been filed or under review. Applications for anti-β-amyloid therapies are under review in several countries, for example, Australia, Canada, India, Russia, Taiwan and Singapore.

## Conclusion

Genetic, biochemical, animal modelling, fluid biomarker and imaging studies support Aβ as a rational target. Improved execution of recent anti-Aβ immunotherapy demonstrates that consistent Aβ lowering decreases pathological tau and delays cognitive decline. ARIA is a characteristic feature of anti-Aβ therapies that will require carefully monitoring. With slowing of cognitive decline with anti-Aβ therapies, whether benefits outweigh the associated risks of ARIA with the treatment requires thorough review. Since the degenerative features of Alzheimer’s disease are multifaceted, combinatorial treatments alongside anti-Aβ therapies are required to produce large clinical effects and more pronounced clinically meaningful results.

## Data Availability

Data sharing is not applicable to this article as no new data were created or analysed in this study.

## References

[1] Knopman DS, Amieva H, Petersen RC, et al Alzheimer disease. Nat Rev Dis Primers. 2021;7(1):33. 33986301 10.1038/s41572-021-00269-y PMC8574196

[2] Glenner GG, Wong CW. Alzheimer’s disease and Down’s syndrome: Sharing of a unique cerebrovascular amyloid fibril protein. Biochem Biophys Res Commun. 1984;122(3):1131–1135. 6236805 10.1016/0006-291x(84)91209-9

[3] Masters CL, Simms G, Weinman NA, Multhaup G, McDonald BL, Beyreuther K. Amyloid plaque core protein in Alzheimer disease and Down syndrome. Proc Natl Acad Sci U S A. 1985;82(12):4245–4249. 3159021 10.1073/pnas.82.12.4245 PMC397973

[4] Hardy J, Selkoe DJ. The amyloid hypothesis of Alzheimer’s disease: Progress and problems on the road to therapeutics. Science. 2002;297(5580):353–356. 12130773 10.1126/science.1072994

[5] Jack CR Jr., Knopman DS, Jagust WJ, et al Tracking pathophysiological processes in Alzheimer’s disease: An updated hypothetical model of dynamic biomarkers. Lancet Neurol. 2013;12(2):207–216. 23332364 10.1016/S1474-4422(12)70291-0 PMC3622225

[6] Sperling RA, Aisen PS, Beckett LA, et al Toward defining the preclinical stages of Alzheimer’s disease: Recommendations from the National Institute on Aging-Alzheimer’s Association workgroups on diagnostic guidelines for Alzheimer’s disease. Alzheimers Dement. 2011;7(3):280–292. 21514248 10.1016/j.jalz.2011.03.003 PMC3220946

[7] Albert MS, DeKosky ST, Dickson D, et al The diagnosis of mild cognitive impairment due to Alzheimer’s disease: Recommendations from the National Institute on Aging-Alzheimer’s Association workgroups on diagnostic guidelines for Alzheimer's disease. Alzheimers Dement. 2011;7(3):270–279. 21514249 10.1016/j.jalz.2011.03.008 PMC3312027

[8] Vermunt L, Sikkes SAM, van den Hout A, et al Duration of preclinical, prodromal, and dementia stages of Alzheimer’s disease in relation to age, sex, and APOE genotype. Alzheimers Dement. 2019;15(7):888–898. 31164314 10.1016/j.jalz.2019.04.001 PMC6646097

[9] Petersen RC, Lopez O, Armstrong MJ, et al Practice guideline update summary: Mild cognitive impairment: Report of the Guideline Development, Dissemination, and Implementation Subcommittee of the American Academy of Neurology. Neurology. 2018;90(3):126–135. 29282327 10.1212/WNL.0000000000004826 PMC5772157

[10] Ward A, Tardiff S, Dye C, Arrighi HM. Rate of conversion from prodromal Alzheimer’s disease to Alzheimer’s dementia: A systematic review of the literature. Dement Geriatr Cogn Dis Extra. 2013;3(1):320–332. 24174927 10.1159/000354370 PMC3808216

[11] Canevelli M, Grande G, Lacorte E, et al Spontaneous reversion of mild cognitive impairment to normal cognition: A systematic review of literature and meta-analysis. J Am Med Dir Assoc. 2016;17(10):943–948. 27502450 10.1016/j.jamda.2016.06.020

[12] 2023 Alzheimer’s disease facts and figures. Alzheimers Dement. 2023;19(4):1598–1695. 36918389 10.1002/alz.13016

[13] Gustavsson A, Norton N, Fast T, et al Global estimates on the number of persons across the Alzheimer’s disease continuum. Alzheimers Dement. 2023;19(2):658–670. 35652476 10.1002/alz.12694

[14] Hong W, Wang Z, Liu W, et al Diffusible, highly bioactive oligomers represent a critical minority of soluble Abeta in Alzheimer’s disease brain. Acta Neuropathol. 2018;136(1):19–40. 29687257 10.1007/s00401-018-1846-7 PMC6647843

[15] Hanseeuw BJ, Betensky RA, Jacobs HIL, et al Association of amyloid and tau with cognition in preclinical Alzheimer disease: A longitudinal study. JAMA Neurol. 2019;76(8):915–924. 31157827 10.1001/jamaneurol.2019.1424 PMC6547132

[16] Budd Haeberlein S, Aisen PS, Barkhof F, et al Two randomized phase 3 studies of aducanumab in early Alzheimer’s disease. J Prev Alzheimers Dis. 2022;9(2):197–210. 35542991 10.14283/jpad.2022.30

[17] Mintun MA, Lo AC, Duggan Evans C, et al Donanemab in early Alzheimer’s disease. N Engl J Med. 2021;384(18):1691–1704. 33720637 10.1056/NEJMoa2100708

[18] van Dyck CH, Swanson CJ, Aisen P, et al Lecanemab in early Alzheimer’s disease. N Engl J Med. 2023;388(1):9–21. 36449413 10.1056/NEJMoa2212948

[19] Swanson CJ, Zhang Y, Dhadda S, et al A randomized, double-blind, phase 2b proof-of-concept clinical trial in early Alzheimer’s disease with lecanemab, an anti-Abeta protofibril antibody. Alzheimers Res Ther. 2021;13(1):80. 33865446 10.1186/s13195-021-00813-8 PMC8053280

[20] Sims JR, Zimmer JA, Evans CD, et al Donanemab in early symptomatic Alzheimer disease: The TRAILBLAZER-ALZ 2 randomized clinical trial. JAMA. 2023;330(6):512–527. 37459141 10.1001/jama.2023.13239 PMC10352931

[21] McDade E, Cummings JL, Dhadda S, et al Lecanemab in patients with early Alzheimer’s disease: Detailed results on biomarker, cognitive, and clinical effects from the randomized and open-label extension of the phase 2 proof-of-concept study. Alzheimers Res Ther. 2022;14(1):191. 36544184 10.1186/s13195-022-01124-2 PMC9768996

[22] Cummings J, Apostolova L, Rabinovici GD, et al Lecanemab: Appropriate use recommendations. J Prev Alzheimers Dis. 2023;10(3):362–377. 37357276 10.14283/jpad.2023.30 PMC10313141

[23] Hampel H, Elhage A, Cho M, Apostolova LG, Nicoll JAR, Atri A. Amyloid-related imaging abnormalities (ARIA): Radiological, biological and clinical characteristics. Brain. 2023;146(11):4414–4424. 37280110 10.1093/brain/awad188 PMC10629981

[24] Karran E, De Strooper B. The amyloid hypothesis in Alzheimer disease: New insights from new therapeutics. Nat Rev Drug Discov. 2022;21(4):306–318. 35177833 10.1038/s41573-022-00391-w

[25] Cummings J . Meaningful benefit and minimal clinically important difference (MCID) in Alzheimer’s disease: Open peer commentary. Alzheimers Dement (N Y). 2023;9(3):e12411. 37521521 10.1002/trc2.12411 PMC10372384

[26] Selkoe DJ, Hardy J. The amyloid hypothesis of Alzheimer’s disease at 25 years. EMBO Mol Med. 2016;8(6):595–608. 27025652 10.15252/emmm.201606210 PMC4888851

[27] Ono K, Condron MM, Teplow DB. Structure-neurotoxicity relationships of amyloid beta-protein oligomers. Proc Natl Acad Sci U S A. 2009;106(35):14745–14750. 19706468 10.1073/pnas.0905127106 PMC2736424

[28] Benilova I, Karran E, De Strooper B. The toxic Abeta oligomer and Alzheimer’s disease: An emperor in need of clothes. Nat Neurosci. 2012;15(3):349–357. 22286176 10.1038/nn.3028

[29] Chen ZL, Singh PK, Calvano M, Norris EH, Strickland S. A possible mechanism for the enhanced toxicity of beta-amyloid protofibrils in Alzheimer’s disease. Proc Natl Acad Sci U S A. 2023;120(36):e2309389120. 37639602 10.1073/pnas.2309389120 PMC10483626

[30] Hardy J, Mummery C. An anti-amyloid therapy works for Alzheimer’s disease: Why has it taken so long and what is next? Brain. 2023;146(4):1240–1242. 36797987 10.1093/brain/awad049 PMC10115350

[31] Cummings J, Osse AML, Cammann D, Powell J, Chen J. Anti-amyloid monoclonal antibodies for the treatment of Alzheimer’s disease. BioDrugs. 2024;38(1):5–22. 37955845 10.1007/s40259-023-00633-2 PMC10789674

[32] Dyer O . Donanemab: FDA experts recommend approval of Alzheimer’s drug. BMJ. 2024;385:q1327. 38876494 10.1136/bmj.q1327

[33] Lannfelt L. A light at the end of the tunnel—From mutation identification to a potential treatment for Alzheimer’s disease. Ups J Med Sci. 2023;128. 10.48101/ujms.v128.10316 PMC10710852 38084203

[34] Jaeschke R, Singer J, Guyatt GH. Measurement of health status. Ascertaining the minimal clinically important difference. Control Clin Trials. 1989;10(4):407–415. 2691207 10.1016/0197-2456(89)90005-6

[35] Molnar FJ, Man-Son-Hing M, Fergusson D. Systematic review of measures of clinical significance employed in randomized controlled trials of drugs for dementia. J Am Geriatr Soc. 2009;57(3):536–546. 19187414 10.1111/j.1532-5415.2008.02122.x

[36] Webster L, Groskreutz D, Grinbergs-Saull A, et al Core outcome measures for interventions to prevent or slow the progress of dementia for people living with mild to moderate dementia: Systematic review and consensus recommendations. PLoS One. 2017;12(6):e0179521. 28662127 10.1371/journal.pone.0179521 PMC5491018

[37] Guyatt GH, Osoba D, Wu AW, Wyrwich KW, Norman GR, Clinical Significance Consensus Meeting G . Methods to explain the clinical significance of health status measures. Mayo Clin Proc. 2002;77(4):371–383. 11936935 10.4065/77.4.371

[38] Andrews JS, Desai U, Kirson NY, Zichlin ML, Ball DE, Matthews BR. Disease severity and minimal clinically important differences in clinical outcome assessments for Alzheimer’s disease clinical trials. Alzheimers Dement (N Y). 2019;5:354–363. 31417957 10.1016/j.trci.2019.06.005 PMC6690415

[39] Burback D, Molnar FJ, St John P, Man-Son-Hing M. Key methodological features of randomized controlled trials of Alzheimer’s disease therapy. Minimal clinically important difference, sample size and trial duration. Dement Geriatr Cogn Disord. 1999;10(6):534–540. 10559571 10.1159/000017201

[40] Howard R, Phillips P, Johnson T, et al Determining the minimum clinically important differences for outcomes in the DOMINO trial. Int J Geriatr Psychiatry. 2011;26(8):812–817. 20848576 10.1002/gps.2607

[41] Vellas B, Andrieu S, Sampaio C, Coley N, Wilcock G, European Task Force G. Endpoints for trials in Alzheimer’s disease: A European task force consensus. Lancet Neurol. 2008;7(5):436–450. 18420157 10.1016/S1474-4422(08)70087-5

[42] Cohen S, Cummings J, Knox S, Potashman M, Harrison J. Clinical trial endpoints and their clinical meaningfulness in early stages of Alzheimer’s disease. J Prev Alzheimers Dis. 2022;9(3):507–522. 35841252 10.14283/jpad.2022.41 PMC9843702

[43] Bateman RJ, Smith J, Donohue MC, et al Two phase 3 trials of gantenerumab in early Alzheimer’s disease. N Engl J Med. 2023;389(20):1862–1876. 37966285 10.1056/NEJMoa2304430 PMC10794000

[44] Salvado G, Molinuevo JL, Brugulat-Serrat A, et al Centiloid cut-off values for optimal agreement between PET and CSF core AD biomarkers. Alzheimers Res Ther. 2019;11(1):27. 30902090 10.1186/s13195-019-0478-z PMC6429814

[45] Jagust WJ, Landau SM, Alzheimer’s Disease Neuroimaging I. Temporal dynamics of beta-amyloid accumulation in aging and Alzheimer disease. Neurology. 2021;96(9):e1347–e1357. 33408147 10.1212/WNL.0000000000011524 PMC8055327

[46] Farrell ME, Jiang S, Schultz AP, et al Defining the lowest threshold for amyloid-PET to predict future cognitive decline and amyloid accumulation. Neurology. 2021;96(4):e619–e631. 33199430 10.1212/WNL.0000000000011214 PMC7905788

[47] Dore V, Krishnadas N, Bourgeat P, et al Relationship between amyloid and tau levels and its impact on tau spreading. Eur J Nucl Med Mol Imaging. 2021;48(7):2225–2232. 33495928 10.1007/s00259-021-05191-9 PMC8175299

[48] Dickson SP, Wessels AM, Dowsett SA, et al ‘Time saved’ as a demonstration of clinical meaningfulness and illustrated using the donanemab TRAILBLAZER-ALZ study findings. J Prev Alzheimers Dis. 2023;10(3):595–599. 37357301 10.14283/jpad.2023.50

[49] Monfared AAT, Ye W, Sardesai A, et al A path to improved Alzheimer’s care: Simulating long-term health outcomes of lecanemab in early Alzheimer’s disease from the CLARITY AD trial. Neurol Ther. 2023;12(3):863–881. 37009976 10.1007/s40120-023-00473-w PMC10195966

[50] Monfared AAT, Tafazzoli A, Chavan A, Ye W, Zhang Q. The potential economic value of lecanemab in patients with early Alzheimer’s disease using simulation modeling. Neurol Ther. 2022;11(3):1285–1307. 35718854 10.1007/s40120-022-00373-5 PMC9338185

[51] Insel PS, Weiner M, Mackin RS, et al Determining clinically meaningful decline in preclinical Alzheimer disease. Neurology. 2019;93(4):e322–e333. 31289148 10.1212/WNL.0000000000007831 PMC6669933

[52] Petersen RC, Aisen PS, Andrews JS, et al Expectations and clinical meaningfulness of randomized controlled trials. Alzheimers Dement. 2023;19(6):2730–2736. 36748826 10.1002/alz.12959 PMC11156248

[53] Pang M, Zhu L, Gabelle A, et al Effect of reduction in brain amyloid levels on change in cognitive and functional decline in randomized clinical trials: An instrumental variable meta-analysis. Alzheimers Dement. 2023;19(4):1292–1299. 36043526 10.1002/alz.12768

[54] Pittock RR, Aakre JA, Castillo AM, et al Eligibility for anti-amyloid treatment in a population-based study of cognitive aging. Neurology. 2023;101(19):e1837–e1849. 37586881 10.1212/WNL.0000000000207770 PMC10663008

[55] Liu KY, Villain N, Ayton S, et al Key questions for the evaluation of anti-amyloid immunotherapies for Alzheimer’s disease. Brain Commun. 2023;5(3):fcad175. 37389302 10.1093/braincomms/fcad175 PMC10306158

[56] Ayton S . Brain volume loss due to donanemab. Eur J Neurol. 2021;28(9):e67–e68. 34224184 10.1111/ene.15007

[57] Alves F, Kalinowski P, Ayton S. Accelerated brain volume loss caused by anti-beta-amyloid drugs: A systematic review and meta-analysis. Neurology. 2023;100(20):e2114–e2124. 36973044 10.1212/WNL.0000000000207156 PMC10186239

[58] Roberts BR, Lind M, Wagen AZ, et al Biochemically-defined pools of amyloid-beta in sporadic Alzheimer’s disease: Correlation with amyloid PET. Brain. 2017;140(5):1486–1498. 28383676 10.1093/brain/awx057

[59] Schott JM, Crutch SJ, Frost C, Warrington EK, Rossor MN, Fox NC. Neuropsychological correlates of whole brain atrophy in Alzheimer’s disease. Neuropsychologia. 2008;46(6):1732–1737. 18395233 10.1016/j.neuropsychologia.2008.02.015

[60] Traini E, Carotenuto A, Fasanaro AM, Amenta F. Volume analysis of brain cognitive areas in Alzheimer’s disease: Interim 3-year results from the ASCOMALVA trial. J Alzheimers Dis. 2020;76(1):317–329. 32508323 10.3233/JAD-190623 PMC7369051

[61] Solopova E, Romero-Fernandez W, Harmsen H, et al Fatal iatrogenic cerebral beta-amyloid-related arteritis in a woman treated with lecanemab for Alzheimer’s disease. Nat Commun. 2023;14(1):8220. 38086820 10.1038/s41467-023-43933-5 PMC10716177

[62] Taylor X, Clark IM, Fitzgerald GJ, et al Amyloid-beta (Abeta) immunotherapy induced microhemorrhages are associated with activated perivascular macrophages and peripheral monocyte recruitment in Alzheimer’s disease mice. Mol Neurodegener. 2023;18(1):59. 37649100 10.1186/s13024-023-00649-w PMC10469415

[63] Roher AE, Maarouf CL, Daugs ID, et al Neuropathology and amyloid-beta spectrum in a bapineuzumab immunotherapy recipient. J Alzheimers Dis. 2011;24(2):315–325. 21263194 10.3233/JAD-2011-101809 PMC3172868

[64] Sakai K, Boche D, Carare R, et al Abeta immunotherapy for Alzheimer’s disease: Effects on apoE and cerebral vasculopathy. Acta Neuropathol. 2014;128(6):777–789. 25195061 10.1007/s00401-014-1340-9

[65] Regenhardt RW, Thon JM, Das AS, et al Association between immunosuppressive treatment and outcomes of cerebral amyloid angiopathy-related inflammation. JAMA Neurol. 2020;77(10):1261–1269. 32568365 10.1001/jamaneurol.2020.1782 PMC7309570

[66] Villar-Fincheira P, Sanhueza-Olivares F, Norambuena-Soto I, et al Role of interleukin-6 in vascular health and disease. Front Mol Biosci. 2021;8:641734. 33786327 10.3389/fmolb.2021.641734 PMC8004548

[67] Castro Caldas A, Silva C, Albuquerque L, Pimentel J, Silva V, Ferro JM. Cerebral amyloid angiopathy associated with inflammation: Report of 3 cases and systematic review. J Stroke Cerebrovasc Dis. 2015;24(9):2039–2048. 26163888 10.1016/j.jstrokecerebrovasdis.2015.04.015

[68] Stone JH, Tuckwell K, Dimonaco S, et al Trial of tocilizumab in giant-cell arteritis. N Engl J Med. 2017;377(4):317–328. 28745999 10.1056/NEJMoa1613849

[69] van Rhee F, Wong RS, Munshi N, et al Siltuximab for multicentric Castleman’s disease: A randomised, double-blind, placebo-controlled trial. Lancet Oncol. 2014;15(9):966–974. 25042199 10.1016/S1470-2045(14)70319-5

[70] Dhillon S . Aducanumab: First approval. Drugs. 2021;81(12):1437–1443. 34324167 10.1007/s40265-021-01569-z

[71] Hoilund-Carlsen PF, Revheim ME, Alavi A, Satyamurthy N, Barrio JR. Amyloid PET: A questionable single primary surrogate efficacy measure on Alzheimer immunotherapy trials. J Alzheimers Dis. 2022;90(4):1395–1399. 36278356 10.3233/JAD-220841 PMC9789473

[72] Alavi A, Barrio JR, Werner TJ, Khosravi M, Newberg A, Hoilund-Carlsen PF. Suboptimal validity of amyloid imaging-based diagnosis and management of Alzheimer’s disease: Why it is time to abandon the approach. Eur J Nucl Med Mol Imaging. 2020;47(2):225–230. 31673787 10.1007/s00259-019-04564-5

[73] Hoilund-Carlsen PF, Alavi A. Aducanumab (marketed as Aduhelm) approval is likely based on misinterpretation of PET imaging data. J Alzheimers Dis. 2021;84(4):1457–1460. 34657891 10.3233/JAD-215275

[74] Klunk WE, Wang Y, Huang GF, et al The binding of 2-(4'-methylaminophenyl)benzothiazole to postmortem brain homogenates is dominated by the amyloid component. J Neurosci. 2003;23(6):2086–2092. 12657667 10.1523/JNEUROSCI.23-06-02086.2003 PMC6741999

[75] Khosravi M, Peter J, Wintering NA, et al 18F-FDG is a superior indicator of cognitive performance compared to 18F-florbetapir in Alzheimer’s disease and mild cognitive impairment evaluation: A global quantitative analysis. J Alzheimers Dis. 2019;70(4):1197–1207. 31322568 10.3233/JAD-190220

[76] Silverman DH, Small GW, Chang CY, et al Positron emission tomography in evaluation of dementia: Regional brain metabolism and long-term outcome. JAMA. 2001;286(17):2120–2127. 11694153 10.1001/jama.286.17.2120

[77] Grady CL, Haxby JV, Schlageter NL, Berg G, Rapoport SI. Stability of metabolic and neuropsychological asymmetries in dementia of the Alzheimer type. Neurology. 1986;36(10):1390–1392. 3762951 10.1212/wnl.36.10.1390

[78] Brown AM, Sheu RK, Mohs R, Haroutunian V, Blass JP. Correlation of the clinical severity of Alzheimer’s disease with an aberration in mitochondrial DNA (mtDNA). J Mol Neurosci. 2001;16(1):41–48. 11345519 10.1385/JMN:16:1:41

[79] Jagust W . Mapping brain beta-amyloid. Curr Opin Neurol. 2009;22(4):356–361. 19478666 10.1097/WCO.0b013e32832d93c7 PMC2882160

[80] Mesulam MM . Neuroplasticity failure in Alzheimer’s disease: Bridging the gap between plaques and tangles. Neuron. 1999;24(3):521–529. 10595506 10.1016/s0896-6273(00)81109-5

[81] Engler H, Forsberg A, Almkvist O, et al Two-year follow-up of amyloid deposition in patients with Alzheimer’s disease. Brain. 2006;129(Pt 11):2856–2866. 16854944 10.1093/brain/awl178

[82] Klunk WE, Mathis CA, Price JC, Lopresti BJ, DeKosky ST. Two-year follow-up of amyloid deposition in patients with Alzheimer’s disease. Brain. 2006;129(Pt 11):2805–2807. 17071918 10.1093/brain/awl281

[83] Jack CR Jr., Lowe VJ, Senjem ML, et al 11C PiB and structural MRI provide complementary information in imaging of Alzheimer’s disease and amnestic mild cognitive impairment. Brain. 2008;131(Pt 3):665–680. 18263627 10.1093/brain/awm336 PMC2730157

[84] Mosconi L, McHugh PF. FDG- and amyloid-PET in Alzheimer’s disease: Is the whole greater than the sum of the parts? Q J Nucl Med Mol Imaging. 2011;55(3):250–264. 21532539 PMC3290913

[85] Walsh S, Merrick R, Milne R, Nurock S, Richard E, Brayne C. Considering challenges for the new Alzheimer’s drugs: Clinical, population, and health system perspectives. Alzheimers Dement. 2024;20(9):6639–6646. 39105453 10.1002/alz.14108 PMC11497759

[86] Barnes J, Dickerson BC, Frost C, Jiskoot LC, Wolk D, van der Flier WM. Alzheimer’s disease first symptoms are age dependent: Evidence from the NACC dataset. Alzheimers Dement. 2015;11(11):1349–1357. 25916562 10.1016/j.jalz.2014.12.007 PMC4619185

